# Establishment of an artificial particulate matter-induced lung disease model through analyzing pathological changes and transcriptomic profiles in mice

**DOI:** 10.1038/s41598-023-29919-9

**Published:** 2023-04-12

**Authors:** Dong Im Kim, Mi-Kyung Song, Ji Eun Yuk, Hyeon Jin Seo, Kyuhong Lee

**Affiliations:** 1 Jeonbuk Department of Inhalation Research, Inhalation Toxicology Center for Airborne Risk Factor, Korea Institute of Toxicology, 30 Baekhak1-Gil, Jeongeup, Jeollabuk-Do 56212 Republic of Korea; 2 Department of Human and Environmental Toxicology, University of Science & Technology, Daejeon, 34113 Republic of Korea

**Keywords:** Molecular biology, Environmental sciences, Diseases

## Abstract

Particulate matter (PM), an environmental risk factor, is linked with health risks such as respiratory diseases. This study aimed to establish an animal model of PM-induced lung injury with artificial PM (APM) and identify the potential of APM for toxicological research. APM was generated from graphite at 600 °C and combined with ethylene. We analyzed diesel exhaust particulate (DEP) and APM compositions and compared toxicity and transcriptomic profiling in lungs according to the exposure. For the animal study, C57BL/6 male mice were intratracheally administered vehicle, DEP, or APM. DEP or APM increased relative lung weight, inflammatory cell numbers, and inflammatory protein levels compared with the vehicle control. Histological assessments showed an increase in particle-pigment alveolar macrophages and slight inflammation in the lungs of DEP and APM mice. In the only APM group, granulomatous inflammation, pulmonary fibrosis, and mucous hyperplasia were observed in the lungs of some individuals. This is the first study to compare pulmonary toxicity between DEP and APM in an animal model. Our results suggest that the APM-treated animal model may contribute to understanding the harmful effects of PM in toxicological studies showing that APM can induce various lung diseases according to different doses of APM.

## Introduction

In 2013, particulate matter (PM) was classified as a group 1 carcinogen by the International Agency for Research on Cancer of the World Health Organization^1^. PM is a complex mixture of solid and/or liquid organic and inorganic substances suspended in the air, which includes organic carbon (OC), elemental carbon (EC), polycyclic aromatic hydrocarbons (PAHs), water-soluble organic ions (chloride, nitrate, sulfate, sodium, potassium, and ammonium), and metals in the natural atmosphere^2^. PM fractions differ in their physical and biological properties according to season, region, and sources^3–7^. High concentrations of OC, EC, and PAHs occur during warm seasons in heavy traffic areas and at night^8,9^. In terms of health effects, exposure to PM, including OC, EC, and PAHs in ambient air, induces and exacerbates clinical symptoms of lung, liver, kidney, and heart diseases^8,10–15^. Most research has focused on epidemiological studies, showing a relationship between air pollutants and emergency room visits and hospital admissions. Currently, toxicological investigations, such as in vivo and in vitro studies, are needed to test for potential toxic effects in humans and speculate on related mechanisms. Many researchers have used standard reference material 2975 (SRM2975, diesel exhaust particulate, DEP) as a component of PM to study health risks associated with PM exposure. DEP is emitted by industrial forklifts and includes PAHs, nitro-PAHs, oxygenated PAH derivatives, heterocyclic compounds, aldehydes, and aliphatic hydrocarbons^16,17^. However, there are some limitations to toxicological studies on PM exposure. High amounts of DEP are required to test lung toxicity, such as inhalation toxicity by PM exposure, especially under high concentrations.

This leads to high costs for studies on the long-term effects of PM exposure. Furthermore, among various sources, toxicological studies by PM may be made by focusing on only DEP toxicity.

In this study, we synthesized artificial PM (APM) in a laboratory setting to use instead of diesel exhaust particulates (DEP) for toxicological studies. We analyzed the OC/EC ratio and PAHs between DEP and APM, compared the pathological features of the respiratory system, and performed transcriptomic analysis of lungs following exposure to APM and DEP through the intratracheal route in an in vivo study to assess the potential of APM for use in respiratory toxicological studies.

## Results

### Comparison of OC/EC ratio and PAHs between DEP and APM

**Figure 1:**
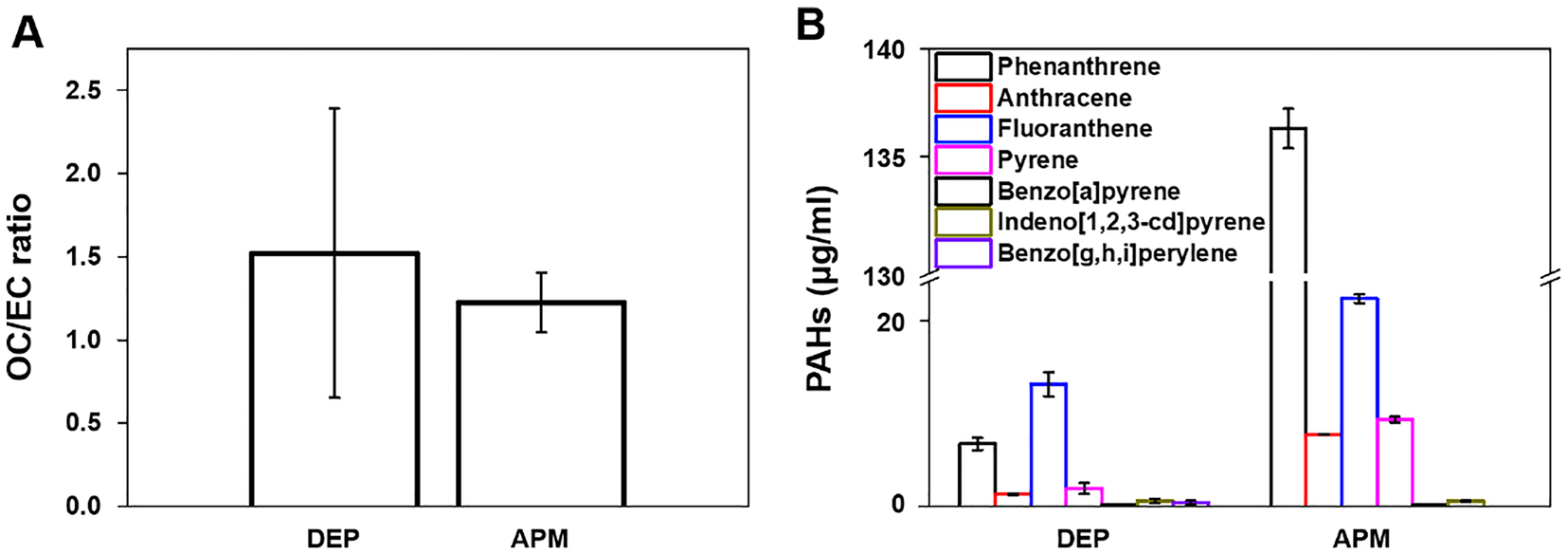
Compositions in DEP and APM. (**A**) OC/EC ratio and (**B**) concentration of PAHs in DEP and APM. Data represent mean ± SD (n = 3). *OC* organic carbon, *EC* elemental carbon, *PAH* polycyclic aromatic hydrocarbons, *DEP* diesel exhaust particulate, *APM* artificial particulate matter.

Our previous study characterized the particle sizes and morphologies of DEP and APM. DEP and APM are generally smaller than 600 nm and 30 nm, respectively^18,19^. DEP is spherical and composed of irregular agglomerates, and APM exhibited typical diesel soot particle morphologies^18,19^. In the present study, we measured the OC/EC ratio and PAHs to identify and compare DEP and APM compositions. The OC/EC ratios of DEP and APM were 1.52 and 1.23, respectively (Fig. 1A). The concentrations of the seven PAHs were measured using DEP and APM. Concentrations of phenanthrene, anthracene, fluoranthene, pyrene, benzo[a]pyrene, indeno [1,2,3-cd] pyrene, and benzo [g,h,i] perylene were 6.42, 1.27, 12.01, 1.56, 0.14, 0.20, and 0.14 μg/mL in DEP and 137.49, 8.00, 22.76, 9.56, 0.17, 0.50, and 0 μg/mL in APM, respectively. The concentrations of phenanthrene, anthracene, fluoranthene, and pyrene were approximately 1.89 to 21.43 times higher in the APM than in DEP.

### Changes in body and organ weights

**Figure 2:**
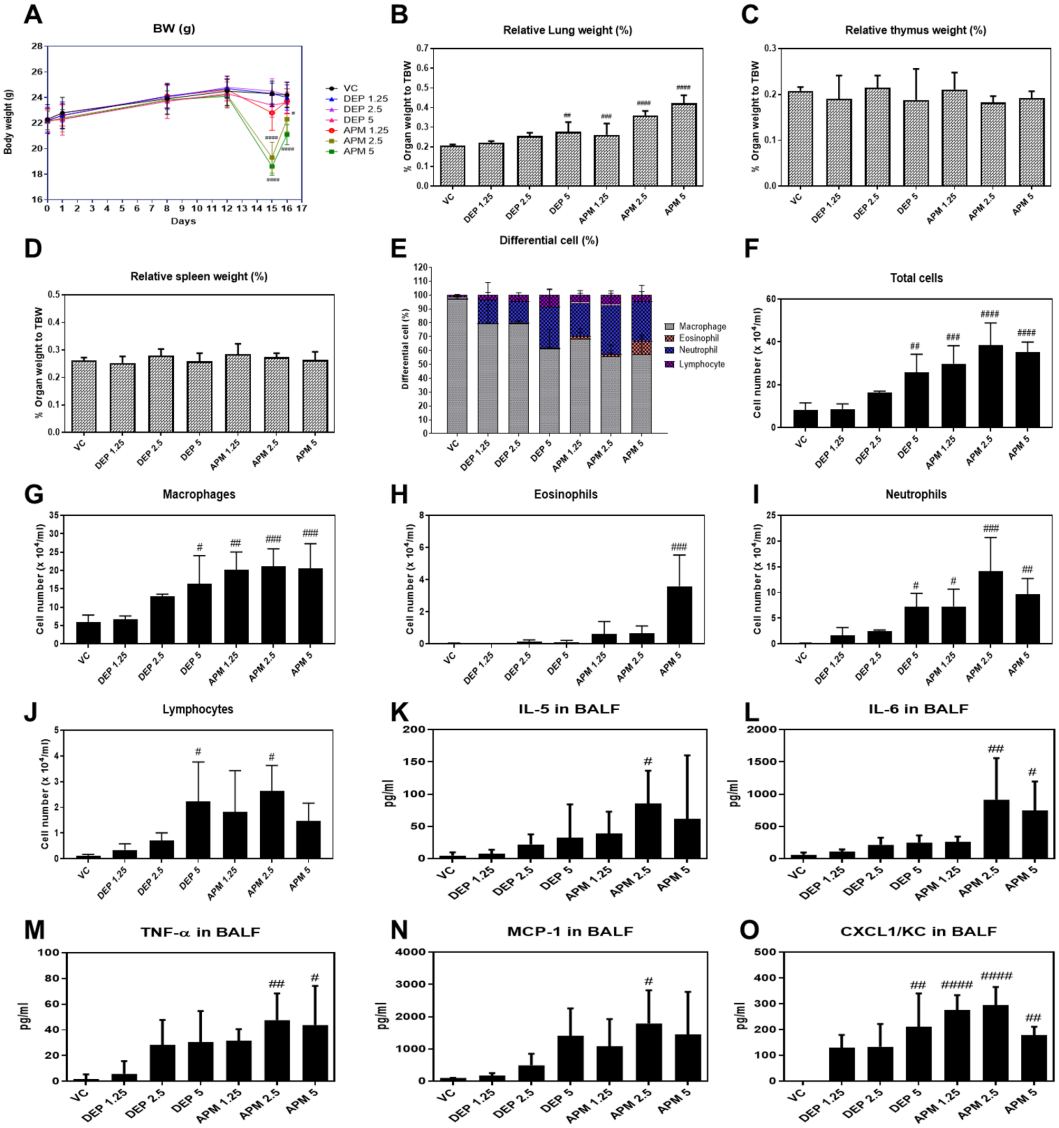
Changes in (**A**) body and (**B**–**D**) relative organ weights, (**E**) cell population, (**F**–**J**) the absolute inflammatory cells, and (**K**–**O**) production of various inflammatory cytokines in the BALF of the vehicle, DEP, and APM-instilled mice. Organ weights were calculated by the following formula: relative organ weight = organ weight (g)/terminal body weight (g) × 100%. Data represent mean ± SD (n = 4 or 5 per group). ^#^p < 0.05, ^##^p < 0.01, ^###^p < 0.001, or ^####^p < 0.0001 vs. VC. *DEP* diesel exhaust particulate, *APM* artificial particulate matter, *BALF* bronchoalveolar lavage fluid, *SD* standard deviation.

There is no change in body weight in VC group. In DEP and APM groups, reduced body weights were observed. Especially, in APM groups, there are significant reduction of body weights (Fig. 2A). Relative lung weight increased statistically in a dose-dependent manner in the DEP and APM groups as compared to that in the VC group (Fig. 2B). The relative lung weight was higher in the APM group than in the DEP group. However, there were no differences in the relative thymus and spleen weights in the DEP and APM groups compared to VC mice (Fig. 2C,D).

### Changes in proportion and the absolute number of inflammatory cells in BALF

We determined whether DEP and APM caused lung inflammation and compared the proportion and number of inflammatory cells in BALF after DEP and APM exposure. First, we measured the proportion of inflammatory cells in the BALF of mice. Macrophages were primarily observed in the control group, representing 97.75% of the total cells. However, a decrease in the proportion of macrophages and an increase in that of neutrophils were commonly observed in the DEP and APM groups. Additionally, the proportions of eosinophils and lymphocytes increased slightly upon exposure to DEP and APM. In particular, the proportion of eosinophils was higher in the APM group than in the DEP group at all doses (Fig. 2E). These results indicated that DEP and APM induced cellular changes and inflammatory responses in the lungs.

Second, the absolute number of inflammatory cells was calculated by dividing the proportion of inflammatory cells by the number of total cells. Relative to the control group, the number of total cells and inflammatory cells such as macrophages and neutrophils, eosinophils, and lymphocytes in BALF increased in the DEP- or APM-instilled groups (Fig. 2F–J). Except for eosinophils, the numbers of total cells, macrophages, neutrophils, and lymphocytes in the BALF of the DEP group gradually increased in a dose-related manner. Although no dose dependency was observed for the increase in the number of total cells and various inflammatory cells in the BALF of the APM group, these were higher in the APM group than in the DEP group at all doses. Particularly, the number of eosinophils significantly increased only in the APM group. These results indicated that although the proportion and number of inflammatory cells in BALF infiltrated by DEP and APM exposure slightly differed, both DEP and APM initiated and maintained inflammatory responses by infiltrating mixed-inflammatory cells in the lungs of mice.

### DEP or APM causes the release of inflammatory cytokines in BALF

We evaluated the levels of various inflammatory cytokines in the BALF of DEP- or APM-instilled mice. DEP and APM induced an increase in the levels of various inflammatory cytokines, such as interleukin (IL)-5, IL-6, tumor necrosis factor (TNF)-α, monocyte chemoattractant protein-1 (MCP-1), and C-X-C motif chemokine receptor 1 (CXCL1)/KC in BALF relative to the VC (Fig. 2K–O). Although there was no statistically significant difference in inflammatory cytokine levels, including those of IL-5, IL-6, TNF-α, and MCP-1, an increasing trend was observed in the DEP group compared to the control group. DEP significantly enhanced only CXCL1/KC levels in the BALF. In the APM group, the inflammatory cytokine levels increased significantly. Consistent with the results of an increase in cellular proportion upon DEP and APM exposure, the release of inflammatory cytokines was stronger in the BALF of the APM group than in the DEP group.

### Histological assessment of the lungs

**Figure 3:**
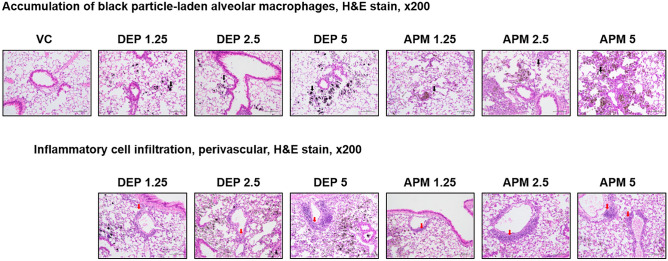
Histological changes in lung tissues upon DEP or APM exposure. Representative H&E-stained slides of lungs obtained from mice instilled with the vehicle, DEP, or APM. Black and red arrows indicate particle-pigment alveolar macrophages and inflammatory cell infiltration, respectively. *DEP* diesel exhaust particulate, *APM* artificial particulate matter, H&E hematoxylin and eosin.

**Figure 4:**
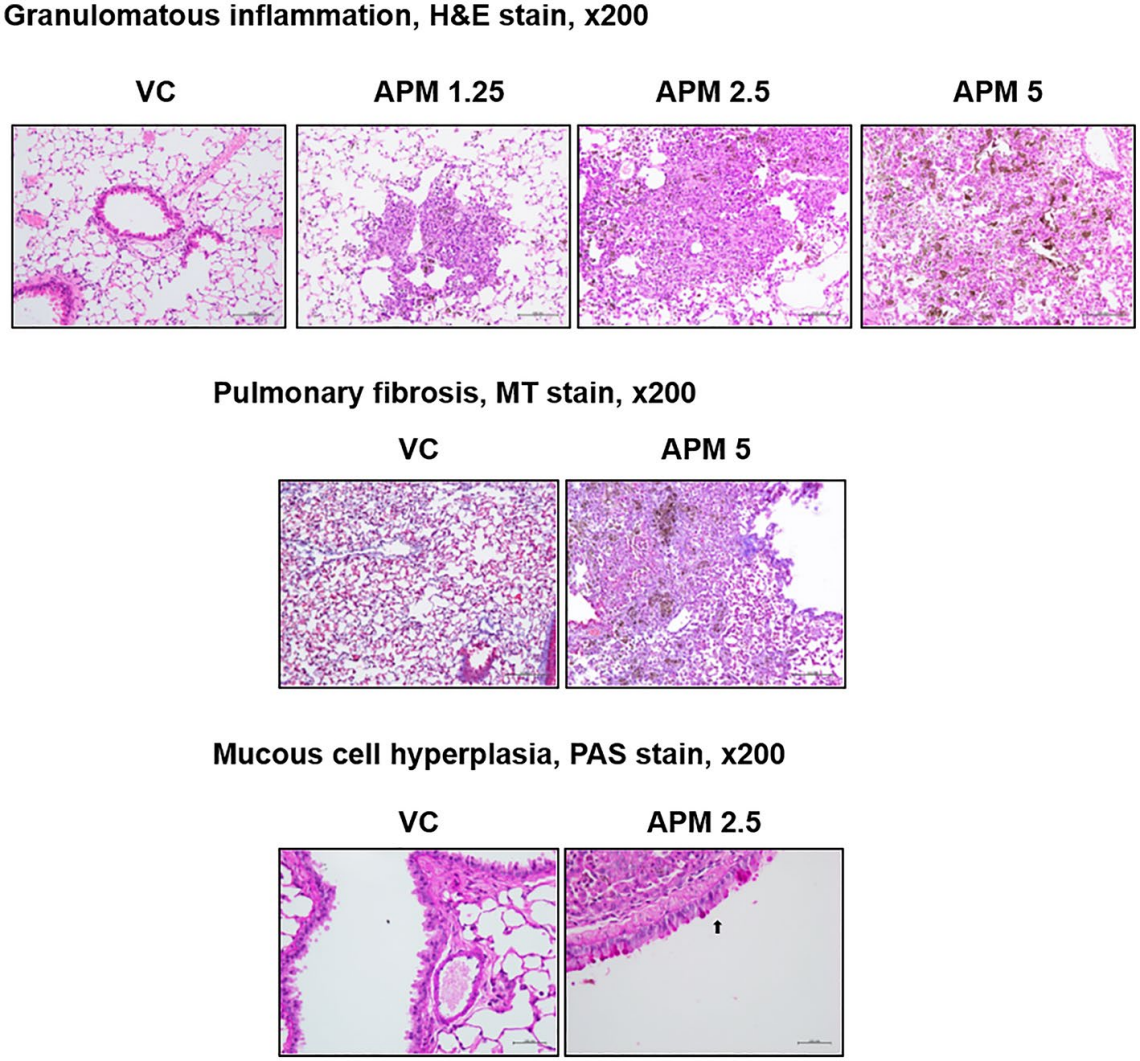
Representative MT- and PAS-stained slides of lungs obtained from mice instilled with vehicle and APM. The black arrow indicates mucous cell hyperplasia. *MT* Masson’s trichrome staining, *PAS* Periodic acid-Schiff, *APM* artificial particulate matter.

Histological assessment via H&E, MT, and PAS staining was conducted to identify and compare the histopathological features in the lung tissues of DEP- and APM-instilled mice. H&E-stained lung sections revealed that DEP and APM instillation commonly resulted in particle-laden alveolar macrophages (black arrows) accumulation in a dose-dependent manner along with slight lung inflammation (red arrows; Fig. 3). In the lungs of some individuals of APM-instilled mice, granulomatous inflammation, pulmonary fibrosis, and mucous cell hyperplasia (Fig. 4) were observed by H&E, MT, and PAS staining, respectively. These findings were confirmed via histological scoring of lung sections (Table 1). These results indicated that both DEP and APM induced alveolar macrophages-mediated acute lung inflammation and APM exposure might lead to more severe lung disease as compared to DEP exposure.

**Table 1:** Quantitative histopathological assessment of staining sections observed in DEP- and APM-instilled mice.

Group	VC	DEP 1.25	DEP 2.5	DEP 5	APM 1.25	APM 2.5	APM 5
Accumulation of particle-pigment alveolar macrophages and black particles in the alveolar lumen							
Minimal	0	1	0	0	0	0	0
Slight	0	4	1	0	1	0	0
Moderate	0	0	2	4	4	5	1
Severe	0	0	0	1	0	0	4
Mean ± SD	0	1.8 ± 0.45^##^	1.6 ± 1.52^##^	3.2 ± 0.45^####^	2.8 ± 0.45^####^	3 ± 0.00^####^	3.8 ± 0.45^####^
Inflammatory cell infiltration, perivascular							
Minimal	0	2	2	1	1	2	0
Slight	0	0	0	0	0	2	2
Moderate	0	0	0	1	0	0	0
Mean ± SD	0	0.4 ± 0.55	0.4 ± 0.55	0.8 ± 1.30	0.2 ± 0.45	1.2 ± 0.84	0.8 ± 1.09
Granulomatous inflammation							
Minimal	0	0	0	0	0	1	2
Slight	0	0	0	0	1	2	2
Moderate	0	0	0	0	0	1	0
Mean ± SD	0	0	0	0	0.4 ± 0.89	1.60 ± 1.14^##^	1.2 ± 0.4^#^
Pulmonary fibrosis							
Minimal	0	0	0	0	0	0	2
Mean ± SD	0	0	0	0	0	0	0.4 ± 0.55
Mucous cell hyperplasia							
Minimal	0	0	0	0	0	*1*	0
Mean ± SD	0	0	0	0	0	0.2 ± 045	0

1, minimal; 2, slight; 3, moderate; 4, severe. Data are presented as mean ± SD from four or five mice per group. *DEP* diesel exhaust particulate matter, *APM* artificial matter. ^#^p < 0.05, ^##^p < 0.01, or ^####^p < 0.0001 vs. VC.

### Comparison of gene expression profiles between DEP and APM exposure

We determined DEP- or APM-altered gene expression profiles and identified Gene ontology (GO) terms to compare the damage-related changes in gene expression induced in response to DEP and APM treatment. Transcriptomic profiling showed differential gene expression patterns in the DEP and APM instillation groups and showed higher sensitivity to gene alterations in the latter than in the former. In total, 257, 341, and 1794 differential expression of genes (DEGs) were identified for DEP exposure, while 849, 1176, and 1842 DEGs were identified for APM exposure at the three corresponding doses. Among these, 40 and 400 genes that were commonly altered at all three doses were identified in DEP and APM exposure, respectively. Among these, 31 and 242 genes showing dose-related expression changes were selected in the DEP and APM groups, respectively. We also identified commonly altered 31 genes in both DEP and APM exposure groups to identify novel biomarkers for PM-altered lung injury. The top 10 genes with the highest fold-changes were *Gpnmb*, *Trem2*, *Clec4d*, *Slc26a4*, *Ear6*, *Cxcl5*, *Slc39a2*, *Cd300lf*, *Cxcr1*, and *Nup62-il4i1*. The expression of nine genes (*Gpnmb*, *Trem2*, *Clec4d*, *Slc26a4*, *Ear6*, *Cd300lf*, *Ctsd*, *Ptgir*, and *Tacc3*) was higher in the APM group than in the DEP group. The genes statistically modulated in the DEP and APM groups were classified through GO biological processes (BPs), Kyoto Encyclopedia for genes and genomes (KEGG) pathways, and disease analysis to analyze the molecular mechanisms related to PM exposure. Key BPs and KEGG pathways that were statistically changed by DEP and APM (Fisher’s exact test p-value < 0.05) are shown in Table 2. Overall, 17 BPs and 5 KEGG pathways were statistically enriched in the DEP exposure groups, and 99 BPs and 15 KEGG pathways were statistically enriched in the APM exposure groups. GO analysis showed that APM-altered genes were linked to more BPs and pathways than the DEP-altered genes. DEP-altered genes were primarily linked to inflammatory responses, including positive regulation of the Extracellular signal-regulated kinase (ERK)1 and ERK2 cascade, response to tumor necrosis factor, and chemokine signaling pathway (Table 2). APM-altered genes were related to inflammatory responses, phagocytic processes, and pro-fibrotic processes due to their involvement in the regulation of various signaling pathways, including positive regulation of ERK1 and ERK2 cascade, positive regulation of NF-kappaB (NF-κB) transcription factor activity, positive regulation of protein kinase B signaling, positive regulation of mitogen-activated protein kinase (MAPK) cascade, cellular response to tumor necrosis factor, positive regulation of phagocytosis, positive regulation of smooth muscle cell proliferation, cellular response to fibroblast growth factor stimulus, and Toll-like receptor signaling pathway (Table 2).

**Table 2:** GO functional category analysis of DEP- or APM-altered genes.

Term	Count	%	p-value	Genes
A. BPs of DEP				
Inflammatory response	6	20	1.15E−04	*Ptgir*, *Vnn1*, *Ccl22*, *Cxcl1*, *Cd14*, *Cxcl5*
Immune system process	4	13.3	0.017	*Marco*, *Clec4d*, *Cd14*, *Cd300lf*
Chemokine-mediated signaling pathway	3	10	0.003	*Ccl22*, *Cxcl1*, *Cxcl5*
Cell chemotaxis	3	10	0.006	*Ccl22*, *Cxcl1*, *Cxcl5*
Positive regulation of ERK1 and ERK2 cascade	3	10	0.030	*Ccl22*, *Gpnmb*, *Trem2*
Acute inflammatory response	2	6.67	0.024	*Vnn1*, *Cxcl1*
Response to tumor necrosis factor	2	6.67	0.035	*Wfdc21*, *Cd14*
B. KEGG pathway of DEP				
Cytokine-cytokine receptor interaction	5	16.67	0.002	*Ccl22*, *Cxcr1*, *Il12b*, *Cxcl1*, *Cxcl5*
Chemokine signaling pathway	4	13.3	0.009	*Ccl22*, *Cxcr1*, *Cxcl1*, *Cxcl5*
C. BPs of APM				
Immune system process	22	9.13	3.59E−09	*C1qb*, *Cd86*, *C1qa*, *Cd84*, *Clec4n*, *Ly96*, *Ctps*, *Tmem173*, *Lilrb4a*, *Serpina3g*, *Cfp*, *Pirb*, *Adgre1*, *Lat2*, *Lgals3*, *Clec4d*, *Aim2*, *Olr1*, *Irf5*, *Cd300lf*, *C1qc*, *Tlr2*
Inflammatory response	18	7.47	5.15E−07	*Ptgir*, *Slc11a1*, *Ly96*, *Cyba*, *Tnfrsf1b*, *Lipa*, *Ptgs1*, *Ccl9*, *Aim2*, *Clec7a*, *C3ar1*, *Spp1*, *Ccl3*, *Olr1*, *Tspan2*, *Ackr1*, *Tnfrsf26*, *Tlr2*
Positive regulation of angiogenesis	10	4.15	1.10E−05	*Acvrl1*, *Lgals3*, *Lrg1*, *Cxcr2*, *Itgb2*, *C3ar1*, *Kdr*, *Hmox1*, *Ctsh*, *Alox12*
Cell surface receptor signaling pathway	10	4.15	1.01E−03	*Adgre1*, *Cd53*, *Cd274*, *Fcer1g*, *Adgre5*, *Tspan7*, *Cxcr2*, *Tspan2*, *Fcgr2b*, *Tnfrsf1b*
Neutrophil chemotaxis	8	3.32	1.42E−05	*Lgals3*, *Ccl9*, *Fcer1g*, *Cxcr2*, *Itgb2*, *Spp1*, *Ccl3*, *Nckap1l*
Positive regulation of ERK1 and ERK2 cascade	8	3.32	6.19E−03	*Ccl9*, *Fam150a*, *Gpnmb*, *Kdr*, *Ccl3*, *Trem2*, *Cd44*, *Tlr2*
Positive regulation of tumor necrosis factor production	6	2.49	5.80E−04	*Fcer1g*, *Ccl3*, *Ly96*, *Cyba*, *Clu*, *Tlr2*
Positive regulation of NF-kappaB transcription factor activity	6	2.49	8.54E−03	*Tgfbr3*, *Cd84*, *Aim2*, *Itgb2*, *Clu*, *Tlr2*
Collagen catabolic process	5	2.07	1.51E−04	*Ctsk*, *Mmp19*, *Mmp8*, *Ctss*, *Ctsb*
Epithelial cell differentiation	5	2.07	7.41E−03	*Cd63*, *Lgals3*, *Krt14*, *Six1*, *Ctsb*
Cell–matrix adhesion	5	2.07	1.25E−02	*Cd63*, *Col3a1*, *Itgb2*, *Itga2b*, *Hpse*
Positive regulation of smooth muscle cell proliferation	5	2.07	1.36E−02	*C3ar1*, *Hmox1*, *Cyba*, *Alox12*, *Igf1*
Positive regulation of protein kinase B signaling	5	2.07	2.57E−02	*F10*, *Hcls1*, *Igf1*, *Hpse*, *Pik3r5*
Positive regulation of MAPK cascade	5	2.07	3.21E−02	*Cd84*, *Igfbp3*, *Kdr*, *Igf1*, *Tnfrsf1b*
Phagocytosis, engulfment	4	1.66	1.49E−02	*Gsn*, *Fcer1g*, *Trem2*, *Fcgr2b*
Positive regulation of nitric oxide biosynthetic process	4	1.66	1.49E−02	*Itgb2*, *clu*, *klf2*, *tlr2*
Positive regulation of interleukin-6 production	4	1.66	2.19E−02	*Il33*, *Fcer1g*, *Cyba*, *Tlr2*
Macrophage chemotaxis	3	1.24	1.09E−02	*Lgals3*, *Ednrb*, *Ccl3*
Extracellular matrix disassembly	3	1.24	2.62E−02	*Mmp12*, *Mmp19*, *Lcp1*
Response to interferon-gamma	3	1.24	3.57E−02	*Cd86*, *Slc11a1*, *Cxcl16*
B cell homeostasis	3	1.24	3.57E−02	*Nckap1l*, *Bcl2a1a*, *Pirb*
Cellular response to fibroblast growth factor stimulus	3	1.24	4.64E−02	*Nr4a1*, *Sfrp1*, *Cd44*
T cell differentiation involved in immune response	2	0.83	4.52E−02	*Clec4d*, *Fcer1g*
D. KEGG pathway of APM				
Lysosome	14	5.81	0.000	*Cd63*, *Hexb*, *Slc11a1*, *Ctsz*, *Lipa*, *Ctss*, *Gnptab*, *Ctsk*, *Ctsh*, *Cd68*, *Atp6v0d2*, *Ctsd*, *Gla*, *Ctsb*
Phagosome	12	4.98	0.000	*Msr1*, *Tuba1c*, *Tubb6*, *Clec7a*, *Mrc1*, *Itgb2*, *Olr1*, *Cyba*, *Fcgr2b*, *Atp6v0d2*, *Ctss*, *Tlr2*
Toll-like receptor signaling pathway	8	3.32	0.001	*Cd86*, *Ctsk*, *Spp1*, *Ccl3*, *Ly96*, *Irf5*, *Tlr2*, *Pik3r5*

*GO* Gene Ontology, *DEP* diesel particulate matter, *APM* artificial particulate matter, *KEGG* Kyoto Encyclopedia of Genes and Genomes.

Finally, PM-altered genes were further analyzed using the Comparative Toxicogenomics Database (CTD) to elucidate the implications of altered genes in lung diseases and other disorders. Both 31 and 242 genes showing dose-related expression changes in the DEP and APM exposure groups, respectively, were associated with various respiratory tract-related diseases, with a greater number of respiratory diseases associated with APM-altered genes. DEP-altered 31 genes were related to respiratory tract diseases, respiratory hypersensitivity, and bronchial diseases, and APM-altered 242 genes were related to respiratory tract diseases, lung diseases, pneumonia, respiratory tract infections, lung neoplasms, and respiratory tract neoplasms (Table 3). As shown in Tables 2 and 3, the numbers in CTD and annotated genes were higher in the APM group than in the DEP group. In the transcriptomic analysis, our results showed that APM exposure induced the expression of more DEGs than DEP exposure in the lungs of mice, and higher expression of APM induced a higher severity of lung diseases by being involved in various BPs than DEP exposure.

**Table 3:** CTD disease category analysis of DEP- or APM-altered genes.

Disease name	Disease categories	p-value	Corrected p-value	Annotated genes quantity	Annotated genes
A. CTD disease category analysis of DEP					
Respiratory tract disease	Respiratory tract disease	6.19E−05	0.01498	6	*Cd14*, *Clec4d*, *Cxcl1*, *Cxcl5*, *Il12b*, *Ptgir*
Respiratory hypersensitivity	Immune system disease, Respiratory tract disease	7.86E−05	0.01902	3	*Cd14*, *Cxcl1*, *Ptgir*
Bronchial disease	Respiratory tract disease	8.62E−05	0.02086	3	*Cd14, Cxcl1, Ptgir*
B. CTD disease category analysis of APM					
Respiratory tract disease	Respiratory tract disease	2.82E−16	2.43E−13	33	*Acvrl1, Adgre5, Bhlhe41, Birc5, Bst1, Ccl3, Cd274, Clec4d, Col3a1, Csf2rb, Cxcr2, Ednrb, Fth1, Hmox1, Igf1, Igfbp6, Il33, Itgb2, Klf2, Lgals1, Mapt, Mdk, Mmp12, Mmp8, Nnat, Npy, Pllp, Prdx1, Ptgir, Ptgs1, Smc2, Spp1, Tnfrsf1b*
Lung disease	Respiratory tract disease	5.78E−16	4.97E−13	30	*Acvrl1, Adgre5, Bhlhe41, Birc5, Bst1, Ccl3, Cd274, Clec4d, Col3a1, Csf2rb, Cxcr2, Ednrb, Fth1, Hmox1, Igf1, Igfbp6, Il33, Itgb2, Klf2, Lgals1, Mdk, Mmp12, Mmp8, Nnat, Npy, Pllp, Prdx1, Smc2, Spp1, Tnfrsf1b*
Lung neoplasm	Cancer, Respiratory tract disease	1.33E−06	0.00114	15	*Bhlhe41, Birc5, Capg, Ccl3, Cd274, Fth1, Hmox1, Lgals1, Mdk, Nnat, Pllp, Prdx1, Smc2, Spp1, Top2a*
Respiratory tract neoplasm	Cancer, Respiratory tract disease	1.46E−06	0.00126	15	*Bhlhe41, Birc5, Capg, Ccl3, Cd274, Fth1, Hmox1, Lgals1, Mdk, Nnat, Pllp, Prdx1, Smc2, Spp1, Top2a*
Respiratory tract infection	Respiratory tract disease	2.31E−07	1.99E−04	9	*Ccl3, Cxcr2, Hmox1, Il33, Itgb2, Mmp8, Npy, Spp1, Tnfrsf1b*
Pneumonia	Respiratory tract disease	2.13E−07	1.83E−04	7	*Ccl3, Cxcr2, Hmox1, Il33, Itgb2, Spp1, Tnfrsf1b*

*DEP* diesel particulate matter, *APM* artificial particulate matter.

## Discussion

PM, a complex mixture of solids and/or liquid organic and inorganic substances, is considered an environmental risk factor. Accumulating evidence suggests that PM is linked to respiratory diseases, including COPD, asthma, fibrosis, and even cancers. Currently, to study the health risks of PM exposure in the lung, many researchers have used DEP as it is difficult to obtain reproducible scientific results due to the diversity of PM by season, region, and source. Moreover, it also takes a long time to collect natural PM to investigate its toxicity in animal and cell studies. However, due to the high costs associated with DEP, toxicological information, including inhalation toxicity under high concentrations for a long time, may be limited. Also, excluding various other factors, only the toxicity of DEP may be investigated. Therefore, to overcome these limitations associated with DEP, we generated APM, analyzed its components, and compared the respiratory effects in DEP- and APM-instilled mice to confirm its applicability to toxicological research. Moreover, we performed a transcriptomic analysis to explain the pathological changes caused by DEP and APM exposure in mice.

We selected DEP, which has been widely used in toxicological studies, as a major PM2.5 component. In previous study, we compared particle size and morphologies between DEP and APM. APM is smaller than DEP size^18,19^. DEP and APM are spherical and composed of irregular agglomerates, and typical diesel soot particle morphologies, respectively^18,19^.

Firstly, in this study, we analyzed the PAHs and OC/EC ratios in APM and DEP. PAHs and OC/EC ratios are associated with the induction and exacerbation of lung diseases^8–15^. Our results showed that PAH levels were higher in APM than in DEP, and the OC/EC ratio was similar between DEP and APM (Fig. 1). PAHs emitted from anthropogenic sources in the ambient air are classified as either carcinogenic or likely carcinogenic to humans in the IARC. Ambient PAHs are associated with the development of lung cancer^20–24^ and non-carcinogenic effects such as decreased lung function, exacerbated asthma, and increased morbidity and mortality of obstructive lung diseases^25–27^. We hypothesized that APM could cause lung diseases with greater severity than DEP, based on particle size and PAHs ratio. In this study, we conducted an animal study to identify and compare the respiratory effects of DEP by measuring body weights during the experimental period, organ weights, cellular changes, levels of inflammatory cytokines in BALF, and histological changes in the lungs of DEP- or APM-instilled mice. We have previously shown that DEP induced neutrophilic dominant lung inflammation in a 100 μg/mouse (5 mg/kg) DEP-instilled group^28–30^. Based on this, the highest dose of DEP and APM was selected as 5 mg/kg. In this study, the mice were intratracheally exposed to DEP and APM. In the DEP- or APM-instilled group, significantly increased relative lung weight was observed compared to that in the VC group (Fig. 2), Increased lung weight indicates an increased vascular permeability and inflammatory cell infiltration into injured lung sites^31^. In this study, although eosinophils were statistically infiltrated in BALF of the only APM group, the proportion of various inflammatory cells such as macrophages, neutrophils, and lymphocytes increased significantly in the BALF of both DEP- and APM-instilled mice (Fig. 2). These results may be related to the increased lung weights of DEP- or APM-instilled mice. We also checked various inflammatory proteins in the BALF of DEP- and APM-instilled mice. ELISA showed that the inflammatory cytokine levels such as IL-6, TNF-α, MCP-1, and CXCL1/KC in BALF were increased in the BALF of both DEP- and APM-instilled mice. These protein levels were particularly higher in the BALF of APM-exposed than DEP-exposed mice (Fig. 2). The protein level of IL-5 was gradually increased in the BALF of DEP groups without any statistically significant change, while this level was increased significantly only in the APM group. MCP-1 is a key chemokine that plays a crucial role in regulating the migration and recruitment of monocytes/macrophages^32^. IL-6 and TNF-α are released from PM-pigmented alveolar macrophages and participate in inflammatory responses^33^. MCP-1, IL-6, and TNF-α are key chemokines or cytokines involved in lung diseases, including asthma and COPD. The chemokine, CXCL1/KC, plays a crucial role in acute inflammation as it is the main chemoattractant responsible for neutrophil recruitment. IL-5 produced from CD4^+^ Th2 cells, mast cells, eosinophils, and basophils are the most potent modulator of eosinophil accumulation during allergic responses such as asthma^34^. Consistent with these results, in histological assessments, mixed-inflammatory cell infiltration in the perivascular regions was commonly observed in the lungs of the DEP and APM groups (Fig. 3, Table 1). However, granulomatous inflammation, pulmonary fibrosis, and mucous hyperplasia were observed in some mice of the only APM groups (Fig. 4, Table 1). These results indicated that both DEP and APM induced lung inflammation accompanied by infiltration of mixed-inflammatory cells, which may induce more severe lung injury than DEP according to the severity of inflammatory cell infiltration and cytokine levels.

Next, we identified the gene expression patterns in DEP- and APM-exposed lungs to understand injury-associated changes in gene expression and related mechanisms and focused on gene expression and GO analysis to elucidate the common pathological changes and different severities of lung injury in DEP- or APM-instilled mice. We found that the transcriptomic changes caused by APM exposure were much more sensitive than those due to DEP exposure, inducing the expression of 242 and 31 genes, respectively, in a dose-dependent manner. Additionally, we identified 31 common genes annotated by DEP and APM exposure to address pathologic changes commonly observed by DEP or APM exposure in an in vivo model. The top 10 genes with the highest fold-changes among the 31 common genes in the DEP and APM groups were *Gpnmb*, *Trem2*, *Clec4d*, *Slc26a4*, *Ear6*, *Cxcl5*, *Slc39a2*, *Cd300lf*, *Cxcr1*, and *Nup62-il4i1*. Among them, nine genes (*Gpnmb*, *Trem2*, *Clec4d*, *Slc26a4*, *Ear6*, *Cd300lf*, *Ctsd*, *Ptgir*, and *Tacc3*) were upregulated in the APM group relative to the DEP group in a dose-dependent manner. The top nine genes have important roles in lung inflammation, asthma, COPD, pulmonary fibrosis, and lung cancer^35–43^. In this study, we observed common lung inflammation in the DEP and APM groups through histological assessment, and the severity was higher in the APM group relative to the DEP group. Pathological features, including granulomatous inflammation, pulmonary fibrosis, and mucous cell hyperplasia, were observed only in the lungs of the APM group. Therefore, our results indicated that the altered gene expression levels might influence the severity of DEP- and APM-induced lung injury.

Key BPs and KEGG pathways significantly affected by DEP and APM exposure are presented in Table 2. DEP- and APM-altered genes are commonly linked to immune and inflammatory responses, including oxidation–reduction processes, angiogenesis, positive regulation of the ERK1 and ERK2 cascade, chemotaxis, and cellular response to tumor necrosis factor. These changes in gene expression are consistent with the results of the cellular changes and cytokine analysis in BALF, showing an increase in macrophages, neutrophils, eosinophils, lymphocytes, and various cytokine levels, including IL-5, IL-6, TNF-α, MCP-1, and CXCL1 (Fig. 2). Genes were further analyzed using the CTD to elucidate gene-disease relationships altered by DEP and APM instillation within the respiratory tract disease category. In a dose-dependent manner, 31 common genes in the DEP groups were involved in various respiratory tract-related diseases (i.e., respiratory tract diseases, respiratory hypersensitivity, and bronchial diseases; Table 3), and 242 common genes in the APM groups were related to various respiratory tract-related diseases (i.e., respiratory tract diseases, lung diseases, pneumonia, respiratory tract infections, lung neoplasms, and respiratory tract neoplasms; Table 3). Similar to the pathological features observed in DEP- or APM-instilled mice, the altered genes observed in the APM group were involved in more BPs and diseases as compared to DEP. These results indicated that APM exposure is more sensitive to gene expression changes related to lung injury than DEP exposure.

Additionally, we analyzed and compared the key BPs between DEP and APM exposure, focusing on asthma because asthmatic features, including eosinophil infiltration and increased IL-5 production, were significantly enhanced in the BALF of the APM group. Besides, mucous hyperplasia was observed in the lungs of the APM group but not the DEP group. Asthma occurs due to genetic and environmental factors such as smoking, various allergens, and pollutants^44^ and is a chronic lung disease characterized by infiltration of eosinophils, enhanced production of Th2 cytokines, increased immunoglobulin E (IgE) production from B cells, increased blood vessels, increased bulk of airway smooth muscle, and excessive mucus production in airways^45,46^ through multiple pathways including MAPK and NF-κB^46,47^. In transcriptomic profiling, by comparing the key BPs between DEP and APM groups, representative asthma indicators, including inflammatory responses, chemotaxis, positive regulation of smooth muscle cell proliferation, positive regulation of nitric oxide biosynthetic process, positive regulation of endothelial cell differentiation, response to Interferon-γ, T cell differentiation involved in immune responses through positive regulation of ERK1 and ERK2 cascade, positive regulation of protein kinase B signaling, and positive regulation of MAPK cascade were identified only in the APM groups. These results showed that APM exposure could induce asthmatic features in the transcriptomic analysis and pathological results from in vivo studies.

Finally, in our study, we generated APM of about 30 nm, which belongs to fine particles as diameter of less than 100 nm. They includes large proportion in atmosphere. In Tiangin of China during 2018 early summer, fine particles constituted 33% of the ambient PM, on average^48^. The European Union has revealed that 70–80% of atmosphere particles was a small diameter of below 100 nm^49^. When small PM was exposed into lungs, they were deposited from pharynx to alveoli, because respiratory track is size-dependent^50^. Smaller size induces risk of respiratory system, cardiovascular system, and all organs because they can pass through capillaries easily^50^. We confirmed these fine particles in lung, heart, kidneys, and spleen due to small size of APM after APM instillation into lungs (not shown) in another study. Therefore, our APM might be suitable and useful to investigate adverse effects of fine particles including initiation, development, and exacerbation of diseases including lung and other organs and related mechanisms.

The current study has a few limitations. First, it is necessary to analyze the composition of APM and compare it with the composition of PM in the air, to establish an animal model reflecting various components of PM and use it for toxicological studies. Second, in this study, DEP and APM treated into an intratracheal route. This method is an alternative to inhalation and it may reveal a different distribution in the lungs. Therefore, further study on inhalation is needed to test the PM toxicity and demonstrate its mechanism of action in PM-inhaled lung injury or other disorders using APM.

Despite these limitations, current observations have revealed the APM-exposed animal model might help study the health adverse effects of PM on the lungs by showing that APM can induce various lung diseases through regulating doses.

## Conclusions

This study established an animal model of PM-induced lung diseases using laboratory-generated APM. Our results showed that both DEP and APM induced inflammatory lung injuries. However, the severity was higher in the APM group relative to the DEP group. In lung of DEP-exposed mice, only slight lung inflammation was observed. Interestingly, APM induced various lung diseases including slight lung inflammation, granulomatous inflammation, pulmonary fibrosis, and asthma according to different doses of APM in mice. Consistent with these results, transcriptomic gene profiling showed that APM-altered genes were involved in more injury-related BPs, pathways, and diseases than DEP. Therefore, we suggest that an APM-instilled animal model has the potential for studying the molecular biological mechanism of PM-induced lung diseases according to various concentration changes, helping develop preventive and therapeutic strategies against PM-related lung diseases.

## Methods

### Animals

Seven-week old male C57BL/6 mice (Orient Bio, Seongnam, Korea) weighing 20.31 ± 0.67 g were maintained in controlled temperature (22 ± 3 °C) and humidity (50 ± 20%) with a 12 h light/12 h dark cycle (150–300 Lux) and free access to food and tap water. Single-housed mice acclimatized for 3 days and showed no adverse clinical signs or normal weight gain. All animal studies were approved by the Institutional Animal Care and Use Committee (IACUC) of the Korea Institute of Toxicology (no. 2105-0030 and no. 2108-0032) and conducted in accordance with ARRIVE guidelines^51^.

### Study protocol

Mice were weight-matched and randomly separated into seven groups (n = 4 for vehicle control or n = 5 for other groups) using Pristima v.7.3 software program for preclinical study (Xybion Medical Systems Corporation, Morris Plains, NJ, USA). The seven groups were defined by specific exposure regimes as follows: vehicle control (VC) for distilled water (DW) as the DEP and APM control; DEP (SRM 2975; National Institute of Standards and Technology, Gaithersburg, MD, USA) for each of the three doses (1.25, 2.5, and 5 mg/kg), and APM for the three doses (1.25, 2.5, and 5 mg/kg). At 24 h after the last instillation to the vehicle, DEP, or APM, measurements of organ weights, analysis of cellular changes and cytokine levels in bronchoalveolar lavage fluid (BALF), and histological examination along with transcriptomic analysis of lung tissue were performed.

### APM generation

In our previous study, carbon black-based APM was produced from graphite at different ranges of temperatures such as room temperature, 200 °C, 400 °C, 600 °C, and 800 °C^18^. In this study, APM was generated from graphite at 600 °C and combined with ethylene to increase in the level of PAHs. Briefly, DNP digital 3000 aerosol generator (Palas GmbH, Germany) was used to generate artificial PM (APM). Under high voltage, a jump spark was generated between two graphite electrodes at 600 °C. After the generator was maintained on a high voltage of 2500 V and 700 Hz between the two graphite electrodes supplying co-mixed gases with pure argon (99.999%; 7 LPM) and air (99.999%; 10 LPM), combined with ethylene. The APM was sampled using Teflon filtered with a pore size of 2 µm and diameter of 47 mm (R2PJ047, PALL Corporation; USA)^18,19^.

### Measurement of OC/EC ratio

Sucrose solutions of varying concentrations were used for equipment calibration (N = 5, R^2^ = 1.00). Quartz fiber filters (QMA filter 25 mm, Whatman Inc., USA) without any organic chemicals were used in APM sampling. The carbonaceous components in APM and DEP were analyzed using an OC-EC Aerosol Analyzer Model 5 (Sunset Laboratory, Beaverton, Oregon, USA)^19^.

### Preparation of PAH standards

Among priority pollutants listed by the United States Environmental Protection Agency, seven PAHs were selected as the targets in this study, including phenanthrene, anthracene, fluoranthene, pyrene, benzo[a]pyrene, indeno[1,2,3-c,d]pyrene, and benzo[g,h,i]perylene. The PAH standards used for calibration and quality assurance were prepared through diluting EPA 610 PAH Mixture (Supelco, St. Louis, MO, USA) with methanol^52^.

### PAH extraction and gas chromatography-mass spectrometry (GC–MS) analysis

Particulate PAHs and n-alkanes were extracted by ultrasonication with a dichloromethane solvent twice for 30 min. The extracts were purified using 0.2 μm-pore size syringe filters. The PAH samples in this study were analyzed using a QP-2010 ultra GC instrument (Shimadzu, Japan) connected to a QP2010 ultra MS instrument (Shimadzu, Japan) with a DB-5MS capillary column^52^. Detailed information is provided in Table 4.

**Table 4:** Operational conditions for analyzing particulate PAHs.

[A]. Gas chromatography	
Injection mode	Splitless
Injection temperature	275.0 °C
Carrier gas	He
Carrier gas pressure	100 kPa
Purge flow	3.0 mL/min
Column flow	1.39 mL/min
Column (DB-5MS)	[length (30 m), diameter (0.25 mm), and film thickness (0.25 µm)]
Oven setting	90 °C (1 min) → 10 °C/min → 200 °C (5 min) → 5 °C/min → 250 °C (5 min) → 5 °C/min → 300 °C (15 min)
	Total operating time = 57 min

[B]. Mass spectrometry	
Ionization mode	70 (eV)
Ion source temperature	230 °C
Interface temperature	150 °C
TIC scan range (retention time (h))	178 (phenanthrene (13.49), anthracene (13.64)), 202 (fluorene (19.105), pyrene (20.28)), 252 (benzo(e)pyrene (36.48)), 276 (indeno(1,2,3-c,d)pytene (42.97), benzo(g,h,i)perylene (44.23) m/z

*PAHs* polycyclic aromatic hydrocarbons.

### Preparation of DEP and APM

DEP and APM were dispersed in a concentration of 2 mg/mL using an ultrasonicator probe (VC 505, Vibra-Cell™ Ultrasonic Liquid Processors, Sonics & Materials, Inc., USA) for 30 min.

### DEP and APM instillation

The respiratory tract is the first route of exposure to PM, and intratracheal administration was used to establish a PM-exposed animal model for respiratory toxicological studies. On days 1, 5, 8, 12, and 15, mice were intratracheally administered with DEP or APM (1.25, 2.5, and 5 mg/kg) dispersed in 50 μl DW. For intratracheal administration, mice were anesthetized using inhaled anesthesia. Prior to administration, isoflurane was delivered into animal chamber using small animal portable anesthesia systems (L-PAS-02, LMSKOREA, Inc, Seongnam, Korea) equipped with isoflurane vaporizer. And then, mice were exposed to 2.5% isoflurane delivered in O_2_ (2 L/min) within animal chamber until a sleep-like state was reached. Mice receiving isoflurane anesthesia were taken out from the animal chamber, mice were lied down on board, and administration was performed immediately using an automatic video instillator. After administration, mice moved, fully recovered and transferred to their cage.

### Measurement of organ weights

On day 16, the lungs, spleen, and thymus were weighed in sacrificed mice.

### BALF collection and analysis

At 24 h after the last DEP and APM instillation, the left lung was ligated, and the right lung was slowly lavaged three times using the tracheal tube with 0.7 mL phosphate-buffered saline (PBS). The number of total cells in the BALF was measured using an NC-250 Counter (ChemoMetec, Gydevang, Denmark). To analyze differential cells, cell smears were conducted using Cytospin equipment (Thermo Fisher Scientific, Waltham, MA, USA) and loaded with Diff-Quik solution (Dade Diagnostics, Aguada, Puerto, USA). After washing, slides were mounted. A total of 200 cells were counted per slide. Also, to measure cytokine levels, the BALF was centrifuged at 2000 rpm for 5 min, and the supernatants were collected.

### Histological analysis

At 24 h after the last DEP and APM instillation, removed lung tissues were fixed in 10% (v/v) neutral-buffered formalin. For slide preparation, lung tissues were dehydrated, embedded in paraffin, and cut into 4-μm sections. Before staining, sections were deparaffinized with xylene. These were stained using hematoxylin and eosin (H&E; Sigma-Aldrich), Periodic acid–Schiff (PAS; Sigma-Aldrich), and Masson's trichrome (MT; Sigma-Aldrich) solutions. The stained sections were analyzed under a light microscope (Axio Imager M1; Carl Zeiss, Oberkochen, Germany). The degree of injury was scored^53,54^.

### RNA isolation

Total RNA was extracted with the TRIzol reagent (Invitrogen). RNA quality and quantity were measured on an Agilent 2100 bioanalyzer with the RNA 6000 Nano Chip (Agilent Technologies, Amstelveen, The Netherlands) and ND-2000 Spectrophotometer (Thermo Inc., DE, USA), respectively. RNA samples with an A260/A280 ratio > 1.8 and RNA integrity number (RIN) value > 7 were used for analysis.

### Library preparation and sequencing

First, the library was constructed using the QuantSeq 3′ mRNA-Seq Library Prep Kit (Lexogen, Inc., Austria) per the manufacturer’s instructions to analyze the control and test samples. An oligo-dT primer, including an Illumina-compatible sequence at its 5ʹ end, was hybridized with the 500 ng of total RNA and reverse transcribed. When the RNA template was degraded, second-strand synthesis was initiated by a random primer, including an Illumina-compatible linker sequence at its 5ʹ end. After purifying the double-stranded library through magnetic beads, the library was amplified to add full adapter sequences needed for cluster generation. High-throughput sequencing was conducted as single-end 75 sequencing on the NextSeq 500 platform (Illumina, Inc., USA), with the final library purified by the PCR components.

### Data analysis

In QuantSeq 3′ mRNA-Seq reads aligned by Bowtie2, Bowtie2 indices were either generated from the genome assembly sequence or the representative transcript sequences for alignment to the genome and transcriptome. The alignment files were used for assembling transcripts, estimating their abundance, and detecting the DEGs. DEGs and the RC (read count) data were processed based on counts from unique and multiple alignments through coverage in Bedtools^55^ and on the quantile normalization method through EdgeR within R using Bioconductor^56^, respectively. Database for Annotation, Visualization and Integrated Discovery (DAVID; http://david.abcc.ncifcrf.gov/) and Medline databases (http://www.ncbi.nlm.nih.gov/) were used for gene classification.

### GO terms and pathway analysis

To classify and compare the genes altered by DEP and APM in animal groups with similar lung toxicity patterns, each gene was assigned to proper categories by its main cellular function. To perform transcriptomic analysis, DEGs were defined under a combination of fold-change ≥ 1.5 and p-value < 0.05. DAVID and pathway analysis was used to determine significantly over-represented GO terms and to determine the significant pathways for DEGs from the KEGG database (http://www.genome.jp/kegg/), respectively. Identification of substantial pathway enrichment and the p-values were adjusted using Fisher’s exact test and the BH false discovery rate (FDR) algorithm, respectively. Pathway categories were reported with FDR < 0.05. Additionally, the CTD (http://ctdbase.org) was used to analyze disease-gene interactions by DEP and APM exposure in mice. In this study, genes that were altered upon DEP or APM exposure in a dose-dependent manner were identified and analyzed.

### Statistical analysis

Data were expressed as the mean ± (SD). Statistical analyses were conducted using the GraphPad Prism 8 software (GraphPad Software Inc., San Diego, CA, USA). The data were examined for variance in homogeneity using Brown–Fosythe test or Bartlett's test. Homogeneous data were analyzed using analysis of variance, and statistical comparisons were conducted using the one-way analysis of variance, followed by Dunnett’s multiple comparisons test. A p-value < 0.05 indicated statistical significance.

### Ethical approval and consent to participate

The animal experiments were carried out following the protocols approved by the IACUC of the Korea Institute of Toxicology (no. 2105-0030 and no. 2108-0032) and conducted in accordance with all guidelines and regulations.

## Data Availability

Data used to support the findings of this study are available from the corresponding author upon request.

## Abbreviations

BP: Biological processes
CTD: Comparative toxicogenomics database
DEG: Differential expression of genes
DEP: Diesel exhaust particulate
DW: Distilled water
EC: Elemental carbon
ERK: Extracellular signal-regulated kinase
FDR: False discovery rate
GO: Gene ontology
H&E: Hematoxylin and eosin
IACUC: Institutional animal care and use committee
KEGG: Kyoto encyclopedia for genes and genomes
MAPK: Mitogen-activated protein kinase
MT: Masson's trichrome
NF-κB: NF-kappaB
OC: Organic carbon
PAH: Polycyclic aromatic hydrocarbon
PBS: Phosphate-buffered saline
PM: Particulate matter
RC: Read count
RIN: RNA integrity number
SD: Standard deviation
VC: Vehicle control

## References

[1] International Agency for Research on Cancer (2013). Outdoor Air Pollution a Leading Environmental Cause of Cancer Deaths, IARC Press Releases.

[2] Chow JC, Watson JG, Ruzer L, Harley NH (2013). Chemical analyses of particle filter deposits. Aerosols Handbook: Measurement, Dosimetry, and Health Effects.

[3] Gentner DR (2012). Elucidating secondary organic aerosol from diesel and gasoline vehicles through detailed characterization of organic carbon emissions. Proc. Natl Acad. Sci. USA.

[4] Contini D, Vecchi R, Viana M (2018). Carbonaceous aerosols in the atmosphere. Atmosphere.

[5] Jia YY, Wang Q, Liu T (2017). Toxicity research of PM2.5 compositions in vitro. Int. J. Environ. Res. Public Health.

[6] Yadav IC, Devi NL, Li J, Zhang G (2018). Polycyclic aromatic hydrocarbons in house dust and surface soil in major urban regions of Nepal: Implication on source apportionment and toxicological effect. Sci. Total Environ..

[7] Gu J (2010). Characterization of atmospheric organic carbon and element carbon of PM2.5 and PM10 at Tianjin, China. Aerosol. Air Qual. Res..

[8] Duan X (2016). Dietary intake polycyclic aromatic hydrocarbons (PAHs) and associated cancer risk in a cohort of Chinese urban adults: Inter- and intra-individual variability. Chemosphere.

[9] Ye Z (2017). Summertime day-night differences of PM2.5 components (inorganic ions, OC, EC, WSOC, WSON, HULIS, and PAHs) in Changzhou, China. Atmosphere.

[10] Löndahl J (2012). Experimental determination of the respiratory tract deposition of diesel combustion particles in patients with chronic obstructive pulmonary disease. Part. Fibre Toxicol..

[11] Ito Y (2016). Nanoparticle-rich diesel exhaust-induced liver damage via inhibited transactivation of peroxisome proliferator-activated receptor alpha. Environ. Toxicol..

[12] Chan TC (2018). Long-term exposure to ambient fine particulate matter and chronic kidney disease: A cohort study. Environ. Health Perspect..

[13] Bates JT (2015). Reactive oxygen species generation linked to sources of atmospheric particulate matter and cardiorespiratory effects. Environ. Sci. Technol..

[14] Zheng XY (2015). Association between air pollutants and asthma emergency room visits and hospital admissions in time series studies: A systematic review and meta-analysis. PLoS ONE.

[15] Strosnider HM (2019). Age-specific associations of ozone and fine particulate matter with respiratory emergency department visits in the United States. Am. J. Respir. Crit. Care Med..

[16] Li N, Wang M, Oberley TD, Sempf JM, Nel AE (2002). Comparison of the pro-oxidative and proinflammatory effects of organic diesel exhaust particle chemicals in bronchial epithelial cells and macrophages. J. Immunol..

[17] Li N (2000). Induction of heme oxygenase-1 expression in macrophages by diesel exhaust particle chemicals and quinones via the antioxidant-responsive element. J. Immunol..

[18] Le YT-H (2021). Relationship between cytotoxicity and surface oxidation of artificial black carbon. Nanomaterials.

[19] Chen X, Kim DI, Moon HG, Chu M, Lee K (2022). Coconut oil alleviates the oxidative stress-mediated inflammatory response via regulating the MAPK pathway in particulate matter-stimulated alveolar macrophages. Molecules.

[20] Vena JE (1983). Lung cancer incidence and air pollution in Erie County. N. Y. Arch. Environ. Health.

[21] Bosetti C, Boffetta P, La Vecchia C (2007). Occupational exposures to polycyclic aromatic hydrocarbons, and respiratory and urinary tract cancers: A quantitative review to 2005. Ann. Oncol..

[22] Rota M, Bosetti C, Boccia S, Boffetta P, La Vecchia C (2014). Occupational exposures to polycyclic aromatic hydrocarbons and respiratory and urinary tract cancers: An updated systematic review and a meta-analysis to 2014. Arch. Toxicol..

[23] Singh A (2018). PAH exposure-associated lung cancer: an updated meta-analysis. Occup. Med..

[24] Armstrong B, Hutchinson E, Unwin J, Fletcher T (2004). Lung cancer risk after exposure to polycyclic aromatic hydrocarbons: a review and meta-analysis. Environ. Health Perspect..

[25] Podechard N (2008). Interleukin-8 induction by the environmental contaminant benzo(a)pyrene is aryl hydrocarbon receptor-dependent and leads to lung inflammation. Toxicol. Lett..

[26] Karimi P, Peters KO, Bidad K, Strickland PT (2015). Polycyclic aromatic hydrocarbons and childhood asthma. Eur. J. Epidemiol..

[27] Yang L (2017). Polycyclic aromatic hydrocarbons are associated with increased risk of chronic obstructive pulmonary disease during haze events in China. Sci. Total Environ..

[28] Kim DI, Song MK, Kim SH, Park CY, Lee K (2019). TF-343 alleviates diesel exhaust particulate-induced lung inflammation via modulation of nuclear factor-κB signaling. J. Immunol. Res..

[29] Kim DI, Song MK, Kim HI, Han KM, Lee K (2020). Diesel exhaust particulates induce neutrophilic lung inflammation by modulating endoplasmic reticulum stress-mediated CXCL1/KC expression in alveolar macrophages. Molecules.

[30] Kim DI, Song MK, Lee K (2021). Diesel exhaust particulates enhances susceptibility of LPS-induced acute lung injury through upregulation of the IL-17 cytokine-derived TGF-β1/collagen I expression and activation of NLRP3 inflammasome signaling in mice. Biomolecules.

[31] Parker JC, Townsley MI (2004). Evaluation of lung injury in rats and mice. Am. J. Physiol. Lung Cell. Mol. Physiol..

[32] Deshmane SL, Kremlev S, Amini S, Sawaya BE (2009). Monocyte chemoattractant protein-1 (MCP-1): An overview. J. Interferon Cytokine Res..

[33] Imrich A (2007). Alveolar macrophage cytokine response to air pollution particles: Oxidant mechanisms. Toxicol. Appl. Pharmacol..

[34] Rosenberg HF, Phipps S, Foster PS (2007). Eosinophil trafficking in allergy and asthma. J. Allergy Clin. Immunol..

[35] Kumagai K (2015). Glycoprotein nonmetastatic melanoma B (Gpnmb)-positive macrophages contribute to the balance between fibrosis and fibrolysis during the repair of acute liver injury in mice. PLoS ONE.

[36] Hall SC, Agrawal DK (2017). Increased TREM-2 expression on the subsets of CD11c+ cells in the lungs and lymph nodes during allergic airway inflammation. Sci Rep..

[37] Byers DE (2018). Triggering receptor expressed on myeloid Cells-2 expression tracks with M2-like macrophage activity and disease severity in COPD. Chest.

[38] Graham LM (2012). The C-type lectin receptor CLECSF8 (CLEC4D) is expressed by myeloid cells and triggers cellular activation through Syk kinase. J. Biol. Chem..

[39] Lee JU (2020). Effects of ammonium chloride on ozone-induced airway inflammation: The role of Slc26a4 in the lungs of mice. J. Korean Med. Sci..

[40] Ishida A (2012). Expression of pendrin and periostin in allergic rhinitis and chronic rhinosinusitis. Allergol. Int..

[41] Maghsoudloo M, Azimzadeh Jamalkandi S, Najafi A, Masoudi-Nejad A (2020). Identification of biomarkers in common chronic lung diseases by co-expression networks and drug-target interactions analysis. Mol. Med..

[42] Cao WJ (2016). High expression of cathepsin E is associated with the severity of airflow limitation in patients with COPD. COPD.

[43] Jung CK (2006). Expression of transforming acidic coiled-coil containing protein 3 is a novel independent prognostic marker in non-small cell lung cancer. Pathol. Int..

[44] Reponen T (2012). Infant origins of childhood asthma associated with specific molds. J. Allergy Clin. Immunol..

[45] Barnes PJ (2016). Asthma mechanisms. Medicine.

[46] Boonpiyathad T, Sözener ZC, Satitsuksanoa P, Akdis CA (2019). Immunologic mechanisms in asthma. Semin. Immunol..

[47] Wang G (2006). Antisense oligonucleotides-induced local blockade of T-bet expression leads to airway inflammation in rats. Acta Pharmacol. Sin..

[48] Khan JZ, Sun L, Tian Y, Dai Q, Hu T, Feng Y (2021). Size distribution of ambient particulate matter and its constituent chemical species involving saccharides during early summer in a Chinese megacity. Front. Environ. Sci..

[49] Grebot B (2011). Industrial Emissions Of Nanomaterials And Ultrafine Particles: Final Report.

[50] Kwon H-S, Ryu MH, Carlsten C (2020). Ultrafine particles: Unique physicochemicalproperties relevant to health and disease. Exp. Mol. Med..

[51] Kilkenny C, Browne WJ, Cuthill IC, Emerson M, Altman DG (2010). Improving bioscience research reporting: The arrive guidelines for reporting animal research. PLoS Biol..

[52] Kim YH, Kim KH (2015). A simple methodological validation of the gas/particle fractionation of polycyclic aromatic hydrocarbons in ambient air. Sci Rep..

[53] Renne R (2009). Proliferative and nonproliferative lesions of the rat and mouse respiratory tract. Toxicol. Pathol..

[54] Ashcroft T, Simpson JM, Timbrell V (1988). Simple method of estimating severity of pulmonary fibrosis on a numerical scale. J. Clin. Pathol..

[55] Quinlan AR, Hall IM (2010). BEDTools: A flexible suite of utilities for comparing genomic features. Bioinformatics.

[56] Gentleman RC (2004). Bioconductor: Open software development for computational biology and bioinformatics. Genome Biol..

